# Increased plasma CD14 levels 1 year postpartum in women with pre-eclampsia during pregnancy: a case–control plasma proteomics study

**DOI:** 10.1038/s41387-019-0105-x

**Published:** 2020-01-14

**Authors:** Antigoni Manousopoulou, Fatma S. Abad, Diana J. Garay-Baquero, Brian R. Birch, Bas B. van Rijn, Bashir A. Lwaleed, Spiros D. Garbis

**Affiliations:** 1 Beckman Research Institute at City of Hope, Duarte, CA USA; 2 Faculty of Health Sciences, University of Southampton, Southampton, UK; 3 Clinical and Experimental Sciences Unit, University of Southampton, Southampton, UK; 4 Faculty of Medicine, University of Southampton, Southampton, UK; 5 Division Woman and Baby, WKZ Geboortecentrum, University Medical Centre Utrecht, Utrecht, The Netherlands; 6 Proteome Exploration Laboratory, Division of Biology and Biological Engineering, Beckman Institute, California Institute of Technology, Pasadena, CA USA

**Keywords:** Risk factors, Endocrine system and metabolic diseases

## Abstract

Epidemiological data suggest that pre-eclampsia (PE) is associated with an increased risk of post-delivery metabolic dysregulation. The aim of the present case–control observational study was to examine the global plasma proteomic profile 1 year postpartum in women who developed PE during pregnancy (*n* = 5) compared to controls (*n* = 5), in order to identify a novel predictive marker linking PE with long-term metabolic imbalance. Key findings were verified with enzyme-linked immunosorbent assay (ELISA) in a separate cohort (*n* = 17 women with PE and *n* = 43 controls). One hundred and seventy-two proteins were differentially expressed in the PE vs. control groups. Gene ontology analysis showed that Inflammatory|Immune responses, Blood coagulation and Metabolism were significantly enriched terms. CD14, mapping to the inflammatory response protein network, was selected for verification based on bibliographic evidence. ELISA measurements showed CD14 to be significantly increased 1 year postpartum in women with PE during pregnancy compared to controls [PE group (median ± SD): 296.5 ± 113.6; control group (median ± SD): 128.9 ± 98.5; Mann–Whitney *U* test *p* = 0.0078]. Overall, the identified proteins could provide insight into the long-term disease risk among women with PE during pregnancy and highlight the need for their postpartum monitoring. CD14 could be examined in larger cohorts as a predictive marker of insulin resistance and type II diabetes mellitus among women with PE.

## Introduction

Pre-eclampsia (PE) is a relatively common pregnancy complication, affecting 2–8% of all pregnancies and a leading cause of perinatal and maternal death. The condition is defined by new onset of hypertension (over 140/90 mmHg, measured on two occasions at least 4–6 h apart) that ensues after the 20th week of gestation. Other common clinical manifestations of PE include foetal growth restriction, signs of maternal organ dysfunction and de novo proteinuria (over 300 mg over 24 h)^1^.

Initially, pre-eclampsia that occurred during the first pregnancy was considered to have no long-term adverse effects^2^. However, emerging epidemiological data suggest that pre-eclampsia is associated with a 2–4-fold increased risk of cardiovascular disease^3^. More specifically, women with pre-eclampsia are at risk for chronic hypertension^4^, fatal and non-fatal coronary heart disease, venous thromboembolism and stroke^5^. With regards to metabolic complications, evidence is more scarce. A systematic review and meta-analysis^6^ showed that PE is independently associated with a 2-fold increased risk of type II diabetes mellitus 1–10 years postpartum.

Plasma proteomics refers to the untargeted analysis of the global circulating proteome. Shotgun proteomics is becoming a very important tool in clinical research, as it can provide valuable insight into the pathophysiology of disease but also identify novel disease markers and therapeutic targets^7–9^. A recently published study by Murphy et al.^10^ examined the maternal circulating proteome 6 months postpartum of women with pre-eclampsia (*n* = 12) compared to controls (*n* = 12) using label-free quantitative proteomics. This study reported the identification of only 126 peptides, exhibiting low coverage of the circulating proteome.

**Fig. 1:**
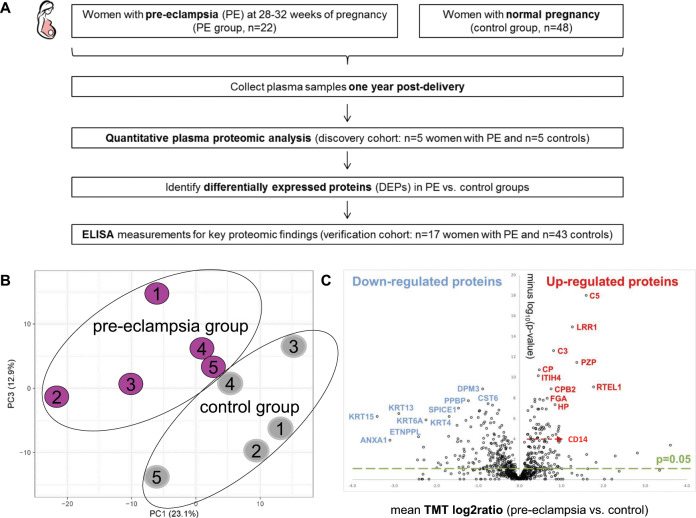
Agnostic Quantitative Proteomics of Non-Depleted Plasma. **a** Study design. **b** Principal component analysis of all analysed plasma proteins. **c** Volcano plot of mean TMT log 2 ratio (PE vs. control) plotted against the minus log 10(*p* value) of the one-sample *T* test for the respective quantified proteins.

The aim of the present study was to examine the global plasma proteomic profile 1 year postpartum in women with pre-eclampsia during pregnancy to identify novel predictive markers of long-term metabolic dysregulation. An overview of the study design is presented in Fig. 1a.

## Materials and methods

### Study participants

Reporting of the present observational study adheres to the STROBE guidelines^11^. The study received ethics approval by the Southampton and South West Hampshire local research ethics committee. Participants were recruited through poster advertising at the Southampton General Hospital and local GP offices between February and April 2017. All participants signed informed consent forms. Females with one child and no miscarriages, who delivered 1 year prior to the recruitment date and either developed or did not develop pre-eclampsia during pregnancy, with normal weight gain during pregnancy (11–16 kg), normal pre-pregnancy and 1-year postpartum body mass index (BMI) (18–25 kg/m^2^) were included in the study. Smokers, females with pre-pregnancy or 1-year postpartum overweight/obesity (BMI > 25 kg/m^2^), pre-pregnancy type I or type II diabetes mellitus, pre-pregnancy hypertension and gestational diabetes were excluded from the study. Seventy participants were considered eligible and all were included in the present case–control observational study (*n* = 48 controls and *n* = 22 women with pre-eclampsia during pregnancy). The study participants were randomly assigned into two cohorts: the discovery cohort (*n* = 5 controls and *n* = 5 women with pre-eclampsia during pregnancy) and the verification cohort (*n* = 43 controls and *n* = 17 women with pre-eclampsia during pregnancy). Plasma from women in the discovery cohort was used for the quantitative proteomics analysis, whereas plasma from women in the validation cohort was used for the targeted enzyme-linked immunosorbent assay (ELISA) validation of key proteomic findings.

### Clinical data analysis

Clinical data were analysed using Prism (version 7.0a). All parameters are presented as mean ± standard deviation. An unpaired, two-tailed Student’s *T* test was applied to compare the baseline characteristics of participants.

### Plasma proteomic analysis

Plasma specimens were subjected to global quantitative proteomic analysis using our previously published method^12–14^. Unprocessed raw files were submitted to Proteome Discoverer 1.4 for target decoy search against the UniProtKB Homo Sapiens database (release date 10 January 2015) using SequestHT. Median normalisation and log 2 transformation were performed for the reporter ion quantification ratios. A protein was considered differentially expressed between the pre-eclampsia vs. control group when its one-sample, two-sided, *T* test *p* value was ≤0.05 and mean tandem mass tag (TMT) log 2 ratio was >|0.3|. Only proteins identified with at least two unique peptides were further considered for bioinformatics analysis. All mass spectrometry proteomics data have been deposited to the ProteomeXchange Consortium via the PRIDE partner repository with the dataset identifier PXD009325.

### Principal component analysis and bioinformatics interrogation

Principal component analysis (PCA) using the reporter ion ratios of the differentially expressed proteins (DEPs) in PE vs. control groups was performed using the online software tool ClustVis (https://biit.cs.ut.ee/clustvis/). DAVID (Database for Annotation, Visualisation and Integrated Discovery; https://david.ncifcrf.gov/) and Ingenuity Pathway Analysis (IPA) (Qiagen, Hilden, Germany) bioinformatics tools were used to identify gene ontology (GO) terms, pathways and protein networks significantly enriched in the DEPs between PE and control groups. Significance was set at *p* value ≤ 0.05.

### Single-blinded ELISA measurements

For the ELISA validation of key proteomic findings, we used a single-blinded design, wherein assignment of participants to PE or control group was not available for the analyst performing the measurements and uncovered by an independent statistician after the measurements were completed. The CD14 ELISA Kit (catalogue no. LS-F4460, Lifespan Biosciences, Seattle, WA, USA) was used as per the manufacturer’s recommendations. Absorbance was measured with the GloMax Discover, Promega plate reader (Thermo Fisher Scientific, Waltham, MA, USA). Data were analysed in Prism (version 7.0a). Statistical analyses of the ELISA measurements were based on the non-parametric Mann–Whitney *U* test to assess differences between the PE and control groups. Significance was set at *p* < 0.05. A receiver-operating characteristic (ROC) curve for CD14 using the ELISA measurements was generated using Prism (version 7.0a). The area under the curve (AUC), standard error (SE), 95% confidence intervals (CIs) and *p* value were also calculated by Prism (version 7.0a).

## Results

For the discovery cohort, PE and control (C) groups were similar with regards to age (PE: 36.3 ± 5.6; C: 34.0 ± 4.0; *p* = 0.47), pre-pregnancy BMI (PE: 22.0 ± 2.6; C: 22.5 ± 2.5; *p* = 0.49), 1-year postpartum BMI (PE: 24.2 ± 3.5; C: 27.0 ± 4.9; *p* = 0.32), 1-year postpartum fasting glucose levels (PE: 4.5 ± 0.8; C: 4.0 ± 0.9; *p* = 0.20), 1-year postpartum fasting insulin levels (PE: 5.0 ± 2.5; C: 4.5 ± 2.0; *p* = 0.31), weight gain during pregnancy (PE: 13.0 ± 2.1; C: 13.5 ± 2.5; *p* = 0.72), gravidity (PE: 1 ± 0; C: 1 ± 0; *p* = 1.0) and parity (PE: 1 ± 0; C: 1 ± 0; *p* = 1.0). Similarly, for the verification cohort PE and control (C) groups were similar with regards to age (PE: 35.4 ± 5.8; C: 34.9 ± 4.4; *p* = 0.58), pre-pregnancy BMI (PE: 23.1 ± 1.8; C: 22.7 ± 2.1; *p* = 0.63), 1-year postpartum BMI (PE: 24.8 ± 2.6; C: 25.2 ± 4.5; *p* = 0.79), 1-year postpartum fasting glucose levels (PE: 4.0 ± 0.8; C: 4.1 ± 1.0; *p* = 0.75), 1-year postpartum fasting insulin levels (PE: 5.3 ± 1.9; C: 4.8 ± 2.6; *p* = 0.62), weight gain during pregnancy (PE: 14.0 ± 3.1; C: 13.0 ± 5.0; *p* = 0.35), gravidity (PE: 1 ± 0; C: 1 ± 0; *p* = 1.0) and parity (PE: 1 ± 0; C: 1 ± 0; *p* = 1.0).

The plasma proteomic study resulted in the identification of 1421 unique proteins (peptide false discovery rate *p* < 0.05). PCA of all quantified proteins showed that women with pre-eclampsia during pregnancy had a distinct plasma proteomic profile 1 year after delivery compared to the control group [data variability incorporated by principal components (PCs) 1, 2 and 3 (%) were 23.1, 13.9 and 12.9, respectively] (Fig. 1b). A volcano plot of the analysed proteome [mean TMT log 2 ratio (PE vs. control) plotted against the minus log_10_ *p* value of the one-sample *T* test per protein] is presented in Fig. 1c.

**Fig. 2:**
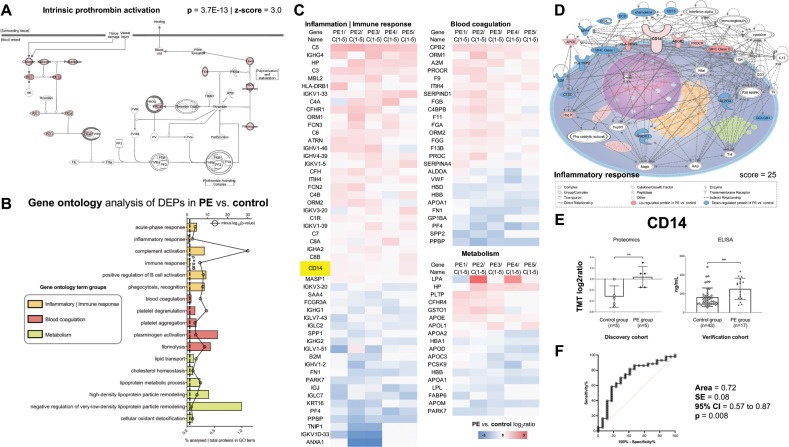
Bioinformatics Interpretation and ELISA Verification. **a** Pathway analysis with IPA showed that the intrinsic prothrombin activation pathway was significantly induced in the PE group compared to control. **b** Gene ontology analysis using DAVID showed that terms related to Blood coagulation, Inflammatory|Immune responses and Metabolism were significantly enriched in the differentially expressed proteins (DEPs). **c** DEPs mapping to these gene ontology term groups in heatmap format. **d** Direct protein interaction network associated with inflammatory response. CD14 mapped onto this network. **e** CD14 was found to be up-regulated 1 year after delivery in women with PE compared to controls with quantitative proteomics. This trend was verified with ELISA in an independent verification cohort. **f** ROC curve using the ELISA measurements of CD14 plasma levels 1 year postpartum. The AUC measure was 0.72, with an SE of 0.08, 95% CI from 0.57 to 0.87, and a *p* value of 0.008. When the cut-off of plasma CD14 concentration was set at 167.6 ng/mL, the sensitivity was 69.77% (95% CI: 54.89–81.40) and specificity 70.59% (95% CI: 46.87–86.72).

In total, 172 proteins were differentially expressed in the PE vs. control groups. The canonical pathway analysis feature of IPA showed that the intrinsic prothrombin activation pathway was significantly induced in the PE group compared to control (Fig. 2a). GO analysis using DAVID showed that terms related to Blood coagulation, Inflammatory|Immune responses and Metabolism were significantly enriched in the DEPs (Fig. 2b). DEPs mapping to these GO term groups are presented in heatmap format in Fig. 2c. IPA showed that the inflammatory response protein network was enriched in the DEPs (Fig. 2d). The monocyte differentiation antigen CD14, which mapped onto the inflammatory response network, was found to be up-regulated 1 year after delivery in women with PE compared to controls [mean TMT log 2 ratio (SD) = 0.3 (4.1)] and this trend was verified with ELISA in an independent cohort [PE group (median ± SD): 296.5 ± 113.6; control group (median ± SD): 128.9 ± 98.5; Mann–Whitney *U* test *p* = 0.0078] (Fig. 2e). To determine the predictive power of CD14 to distinguish between women with pre-eclampsia vs. controls 1 year postpartum, an ROC curve was generated (Fig. 2f). The AUC measure was 0.72, with an SE of 0.08, 95% CI from 0.57 to 0.87 and a *p* value of 0.008. When the cutoff of plasma CD14 concentration was set at 167.6 ng/mL, the sensitivity was 69.77% (95% CI: 54.89–81.40) and the specificity was 70.59% (95% CI: 46.87–86.72).

## Discussion

This study reports the most comprehensive to date plasma proteomics profiling of women with pre-eclampsia compared to controls 1 year postpartum. The study results show that Inflammation|Immune response, blood coagulation and metabolism were dysregulated processes 1 year after delivery in women with pre-eclampsia during pregnancy.

An observational case–control study with a mean follow-up of 3 years that included ~13,000 women in the pre-eclamspia group and 280,000 women in the control group identified a small but significantly increased risk of venous thromboembolism as a result of pre-eclampsia during pregnancy^15^. In our study, the identification of the induction of the intrinsic prothrombin activation pathway (Fig. 2a) and blood coagulation as significantly enriched GO term (Fig. 2b) served as positive controls for the validity of our global plasma proteomic profiling.

The monocyte differentiation antigen CD14 (CD14) mapped to the immune/inflammatory response GO term (Fig. 2c) and the inflammatory response protein interaction network (Fig. 2d). Based on this evidence and bibliographic research, CD14 was selected for further ELISA verification as a potential marker linking PE with long-term metabolic dysregulation. CD14, a 55-kDa protein expressed found in membrane-anchored and soluble serum protein forms, is a co-receptor for bacterial lipopolysaccharide and mediator of the inflammatory response. Furthermore, CD14 has been shown to participate in adipose tissue-related chronic inflammation, and the eventual development of insulin resistance as a result of chronic inflammatory signals^16^. Our plasma proteomic results showed that women with pre-eclampsia 1 year after delivery have higher CD14 levels compared to controls and this finding was verified using ELISA in an independent cohort (Fig. 2e). The ROC curve analysis showed a moderate level of success (71.96% AUC, 95% CI: 56.95–86.96) for using CD14 plasma levels to distinguish women with pre-eclampsia vs. controls 1 year postpartum. This increase in circulating CD14 levels could reflect a pro-inflammatory status of women with pre-eclampsia after delivery and possible underlying risk of developing insulin resistance and type II diabetes mellitus.

The main drawback of the present study is its small sample size. Due to the limited sample size, we expect some of the identified DEPs to be false positives. In conclusion, the present plasma proteomics profiling 1 year postpartum of women with PE vs. controls provides insight into the dysregulated cardiometabolic profile in this population group and highlights the need for their long-term health monitoring. The clinical importance of CD14 as a predictive marker of insulin resistance and type 2 diabetes mellitus among women with PE during pregnancy should be further examined in large multi-centre cohorts.
